# PepBank - a database of peptides based on sequence text mining and public peptide data sources

**DOI:** 10.1186/1471-2105-8-280

**Published:** 2007-08-01

**Authors:** Timur Shtatland, Daniel Guettler, Misha Kossodo, Misha Pivovarov, Ralph Weissleder

**Affiliations:** 1 Center for Molecular Imaging Research, Massachusetts General Hospital, Harvard Medical School, Bldg. 149, 13^th ^Street, Room 5406, Charlestown, MA 02129, USA; 2 Northern Essex Community College, 100 Elliott Street, Haverhill, MA 01830, USA

## Abstract

**Background:**

Peptides are important molecules with diverse biological functions and biomedical uses. To date, there does not exist a single, searchable archive for peptide sequences or associated biological data. Rather, peptide sequences still have to be mined from abstracts and full-length articles, and/or obtained from the fragmented public sources.

**Description:**

We have constructed a new database (PepBank), which at the time of writing contains a total of 19,792 individual peptide entries. The database has a web-based user interface with a simple, Google-like search function, advanced text search, and BLAST and Smith-Waterman search capabilities. The major source of peptide sequence data comes from text mining of MEDLINE abstracts. Another component of the database is the peptide sequence data from public sources (ASPD and UniProt). An additional, smaller part of the database is manually curated from sets of full text articles and text mining results. We show the utility of the database in different examples of affinity ligand discovery.

**Conclusion:**

We have created and maintain a database of peptide sequences. The database has biological and medical applications, for example, to predict the binding partners of biologically interesting peptides, to develop peptide based therapeutic or diagnostic agents, or to predict molecular targets or binding specificities of peptides resulting from phage display selection.  The database is freely available on , and the text mining source code (Peptide::Pubmed) is freely available above as well as on CPAN ().

## Background

Peptides have emerged as important affinity ligands for diagnostic and therapeutic medical uses as well as materials for a host of applications in biotechnology. While many excellent databases exist that provide protein sequence data [1-3], protein interaction data [4-9], and peptide data [10-13], a substantial fraction of literature data remains untapped. Unfortunately, the wealth of the peptide sequences in these sources is often difficult to access by modern methods of sequence similarity searching, because peptide sequences are not extracted in a suitable format. We therefore sought to address this issue by developing a combination of automatically mining MEDLINE abstracts for peptide sequences, combining the existing bioinformatics sources, and manually curating the full text articles and MEDLINE text mining results. The data, available through a web-based interface for simple and more advanced text search and BLAST and Smith-Waterman sequence similarity search, proved useful in our own work. Examination of initial data yielded some surprises as well, providing an incentive for us to make further improvements to the database. We hope that the peptide database, the associated tools, and the text mining algorithm will be useful to the larger biomedical community.

Peptides are defined by International Union of Pure and Applied Chemistry and International Union of Biochemistry and Molecular Biology (IUPAC-IUB) as compounds "produced by amide formation between a carboxyl group of one amino acid and an amino group of another" [14]. In this paper, we use the term "peptides" as a common synonym for oligopeptides, which are defined as having "fewer than about 10–20 residues"[14]. We thus currently use an IUPAC-IUB length cut-off of 20 amino acid residues or less. Many of the peptides used as pharmaceutical and diagnostic agents fall within this cut-off.

Naturally occurring peptides function as hormones, transmitters, and modulators of numerous biological processes [15]. Both naturally occurring and synthetic peptides are used in therapeutic applications [15], for example somatostatin analogs in tumor radiotherapy [16,17] and oxytocin to induce labor [18]. Examples of diagnostic uses include membrane-translocating agents [19], receptor targeting agents [20], and enzyme substrates [21]. Driven by the great interest in the diverse applications of peptides, the new peptidomics field is rapidly emerging [22]. The functions of peptides, including their interacting partners, are determined by their sequence and similar to longer proteins, can be predicted based on sequence similarity.

Prior knowledge can be used to predict or shorten the list of possible binding partners of a given peptide of interest, provided a peptide shares significant sequence similarity with other peptides or proteins whose binding partners are known [20,23]. One can also use a sequence similarity search to remove peptides with similarity to other peptides with known, undesirable properties such as non-specific binding [24] or toxicity. Computational predictions are relatively fast and inexpensive, but require a peptide sequence database with links to peptide data, for use with sequence similarity search methods such as basic local alignment search tool (BLAST) [25,26] or Smith-Waterman search [27,28]. The non-sequence (text) data in such a peptide database can be queried with text search tools for biological, therapeutic or diagnostic applications, for example to find peptides that are enzyme inhibitors and whose sequences are available.

We searched through the existing bioinformatics sources, and found no single source that fully suited our needs. With the exception of the Receptor Ligand Contacts (RELIC) database and web-server [10] and Artificially Selected Proteins/Peptides Database (ASPD) [11], most large protein sequence and interaction databases that allow both sequence similarity and text annotation searches have two major drawbacks. First, most of their sequences are of biological origin, while many phage display [29,30] or combinatorial screens yield non-biological sequence hits. There is no large repository of chemically generated unnatural sequences, similar to what PubChem [2] or ChemBank [31] are for compounds. Second, there exists less data on short peptides than on longer proteins, and usually no facile way to restrict the search to short sequences only. This is important because performing an unrestricted sequence similarity search often results in a large proportion of false positives due to hits to proteins in which the peptide sequence is buried and not accessible for binding, or is in a conformation different from that in a shorter peptide. The same sequence may have different binding properties when displayed on a phage versus when presented as part of the native protein [32]. Sequence similarity based predictions are further hampered for conformationally constrained peptides, designed specifically to have properties different from the same sequence in linear form [33]. ASPD [11] and RELIC [10] databases do not have these drawbacks, are well curated, but are relatively small compared with the large amount of sequence data in the MEDLINE abstracts. For example, the ASPD database has 1,717 entries of 20 amino acid or shorter sequences. RELIC (a server with many useful peptide sequence analysis tools) has 3,632 peptide sequences that result from phage display selections, but only 7 distinct targets to which they bind. Other peptide databases have different purposes and are more specialized by design, for example antimicrobial (the Antimicrobial Peptide Database (APD) [13], and others [12,34,35]), phosphorylation sites (Scansite [36]), or major histocompatibility complex related (SYFPEITHI [37], EPIMHC [38], and others [39-42]).

In order to create a database suitable for the identification of affinity ligands, we developed text mining methods to extract peptide sequences from MEDLINE abstracts and compiled them in a single, easily searchable database. While far from complete, the database is a useful publicly available source of peptide sequences and the associated data. Below we show how the database was constructed, how it functions, and how it can be used to identify targeting ligands.

## Construction and content

### Database model and overview

**Figure 1:**
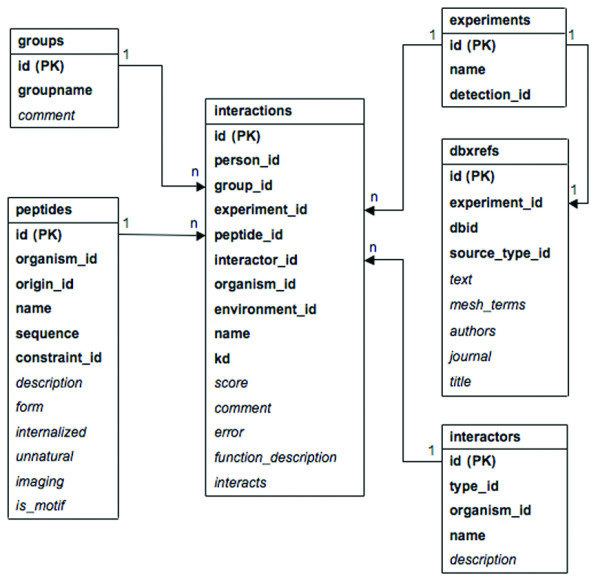
**Database core model**. MySQL tables are shown as rectangles. Mandatory attributes are in **bold**, optional are in *italics*. Relationships are shown as lines, with the arrows pointing from the primary to the foreign keys, and multiplicities as shown.

The database model (Figure 1) was adopted from the Proteomics Standards Initiative Molecular Interactions (PSI MI) model for storage of biological interactions [43] and was extended to facilitate secure access to curate entries. Each entry is associated with a "peptide sequence", an "interactor", an "experiment" and a "group". The group serves to assign user permissions for curating entries. Separate tables, which are not shown for clarity, define controlled vocabularies. These were adopted where possible from the existing ontologies. Organism vocabulary used for peptides, interactors and interactions was adopted from the National Center for Biotechnology Information (NCBI) Taxonomy [2]. The detection method vocabulary, utilized for experiments, was adopted from PSI MI ontology using the descendants of the term MI:0001, "interaction detection method".

The application is using the open source Ruby on Rails framework [44] with a MySQL database [45] in the backend. The BLAST search [25,26] was implemented using the NCBI binaries [46]. The Smith-Waterman search was implemented using the SSEARCH program from the FASTA3 distribution [27,28,47]. The databases for sequence similarity searches included, in addition to sequences, the motifs, with any variable positions replaced with X for simplicity (for example, motif 'P(P/S)GH(Y/F)K' was used as 'PXGHXK').

The database was constructed from the following sources (with the current number of entries in parentheses): text mining of MEDLINE abstracts (13,596 entries), manual curation of full text PDF articles (859), and other public sources: ASPD (1,717) and UniProt (3,620), as described in the sections below. The total number of entries is currently 19,792. A small fraction of the peptide sequences resulting from MEDLINE abstract text mining were manually curated: 1,773 entries were validated as correct peptide sequences, and 170 of those were more fully annotated with additional interaction data present in the abstract. The database continues to grow as the new data are added to the sources such as MEDLINE and UniProt.

### MEDLINE abstract text mining

In order to identify abstracts with peptide sequences, the entire MEDLINE database with its 15 million records was downloaded from the National Library of Medicine (NLM) ftp site [48]. The text mining code was written in Perl, a language selected due to its text processing capabilities, and widely used in many important biomedical literature text mining applications [49-51]. Data were processed in 3 steps. First, each abstract was assigned a score based on how likely it was to contain a peptide sequence anywhere within the text. Second, each individual word was assigned a score based on how likely it was to contain a peptide sequence. For each word, a combined score was then computed based on both the word score and the abstract score. Thus, in total, we used three types of scores (abstract, word, and combined). Third, the sequences associated with the words were cleaned, and ambiguities resolved. After these tasks were completed, the words were ranked by the combined score and included in the peptide database based on empirically determined thresholds. Each unique sequence per abstract identified by text mining was assigned one database entry. Multiple occurrences of the same sequence in different forms, such as 'RGD' and 'Arg-Gly-Asp', were considered a single entry.

Text mining was performed on a Fedora Core 5 Linux virtual machine running on an HP DL320 server with two 3 GHz Xeon processors, allocated 512 MB of RAM. The data resided on a file server connected via Gigabit Ethernet. Text mining of the entire MEDLINE (baseline distribution and updates) took 44 hours, with an additional 16 hours for pre-processing: downloading, uncompressing/compressing and parsing MEDLINE distribution files. The resulting database was 35 MB. Incremental weekly processing of MEDLINE updates took on average under 1 hour.

#### Step 1. Classification of abstracts

MEDLINE entries that were either duplicates, or did not have abstracts, or were older than 1950 were removed. The older abstracts, which were published prior to the development of Edman degradation [52], did not contain peptide sequences. Several pattern categories of interest were created, such as those related to peptides, phage display, proteases, and others. For each abstract, the total number of matches to patterns in each category was computed, for example, for the 'peptide' category this included the number of matches to 'peptid' or 'hormone', and if at least one of these patterns was present, additionally included the number of matches to less specific patterns such as 'sequenc' or 'motif'. The title, abstract, medical subject heading terms and the chemical list were all scored. Some of the abstracts, especially those published before mid-1990s, often include peptide sequences which are related to protein digestion and sequencing. These sequences usually represent parts of longer proteins, rather than individual peptides, and were thus scored differently. Any matches to this 'digestion' category of patterns were counted. The abstract score was computed as the sum of the number of matches to categories 'peptide' and 'phage', minus the number of matches to the 'digestion' category. Additional terms were added to the abstract score for matches to more than one pattern category in the same abstract, for example the number of matches to patterns from the 'phage' category multiplied by the number of matches to the 'peptide' category. Phosphorylated peptides, such as those selected using the oriented phosphopeptide library technique [53], were not scored any differently from other peptides, that is, neither included not excluded specifically. There is a useful resource, Scansite, dedicated specifically to the phosphorylated peptides [36], which can be used for this application. Texts with a large number or fraction of words in all caps tend to produce many false positives, thus the abstract score was decreased for such abstracts. The abstract score was then transformed for convenience to the (0,1) interval using the function: *y = x/(1+x)*. An abstract score below 0 was assigned to 0. An abstract related to peptide sequences tended to have a score close to 1, and an unrelated one to 0.

#### Step 2. Classification of words

Each abstract was split into words on whitespace. Each word was matched against a series of peptide sequence pattern categories, in order of decreasing specificities of patterns, until the first successful match. The pattern categories were: full names of amino acids (longest, most specific, such as 'valine' or 'valyl'), 3 letter symbols (such as 'Val') and 1 letter symbols (such as 'V', least specific). Because the recommendations of IUPAC-IUB for reporting peptide sequences [14] were not followed in a large number of abstracts, we had to use a complex classification method and added methods to clean sequences and resolve the ambiguities. Any word that matched a pattern of peptide sequence of at least two amino acids was assigned a score. The score was an empirically calculated measure used to distinguish peptide sequences from other terms, such as nucleic acid sequences, gene symbols, acronyms and all caps English words, which they sometimes closely resemble or are even identical to, when taken out of context.

The above score was defined by several factors. The length/amino acid symbol factor was based on the length of the sequence in amino acids (higher score for longer sequence patterns, which were more specific) and on the type of amino acid symbols used (higher score for the more specific full names than for 1 letter symbols). The degenerate amino acid factor was based on the fraction and the total number of degenerate amino acids (lower score for degenerate amino acids such as 'X' or 'Xaa', which may represent, for example, the starting randomized phage display library rather than the selected peptide). Other factors reflected similarity to either of the following categories: Roman numerals, nucleic acid sequences, gene names and gene symbols, English words, scientific terms or abbreviations, or a combination of the above. The list of abbreviations was derived from the comprehensive ADAM database [54]. The list of gene names and symbols was derived from Entrez Gene [55], UniProt [1] and Human Gene Nomenclature (HGNC) [56] databases. An additional factor represented similarity of a given word to protein sequences relative to English words. It was computed for all words that matched a pattern of sequences in 1 letter amino acid symbols. The word was broken up into overlapping k-mers. For example, for k = 3, word 'EYHHYNK' was broken up into 'EYH', 'YHH', 'HHY', 'HYN', 'YNK'. The proportions of all possible k-mers were precomputed in the databases of known protein sequences (from UniProt) and non-sequences (here, English words from MEDLINE abstracts not related to peptides), designated P_p _and P_n_, respectively. We used the databases of protein sequences and non-sequences of 8 × 10^7 ^k-mers each, with k = 3, replacing counts of 0 with 1 to avoid division by 0. The protein/English word similarity factor was defined as the product over all overlapping k-mers within the word of (P_p_/P_n_). For a word with all k-mers equally frequent among sequences and non-sequences, the factor was 1, while for a word such as 'EYHHYNK' in which on average the k-mers were more frequent in protein sequences than in English words, the factor was greater than 1.

The word score was transformed to the (0,1) interval, similarly as in the abstract score. The word score thus depended only on the properties of the word itself, rather than on the context (the properties of the abstract). The combined word/abstract score was then computed for each word, and reflected the abstract score, the word score, and the maximum word score for all words in the abstract, included because sequences tend to occur together in abstracts. The combined word/abstract score s_c _was computed according to the formula

s_c _= s_a_(w_1_s_w _+ w_2_s_m_), for s_w _> 0,

s_c _= 0, for s_w _= 0,

where s_a _is the abstract score, s_w _is the word score of the current word, s_m _is the maximum word score for all words in the abstract, and w_1_, w_2 _are the weights (w_1 _> w_2_). The combined score varied in the (0,1) interval. Words that matched peptide sequence patterns in abstracts related to peptides tended to have a score close to 1, and close to 0 otherwise.

#### Step 3. Clean-up

Words that matched peptide sequence patterns were cleaned in a series of steps and converted to 1 letter amino acid symbols, as follows. The terminal marks and modifications, such as 'H(2)N-' or '-CO-Ph', were removed. Numbers representing amino acid positions were removed. Other modifications, such as phosphate in 'pY' were removed. Motifs such as '(L/I)' or 'L/I' were resolved. Amino acids that do not have a 1 letter IUPAC symbol were replaced with X. As a result, a large variety of different sequence formats were resolved, including 'N-acetyl-l-aspartyl-l-glutamyl-l-valyl-l-aspartyl-7-amino-4-methylcoumarin' to 'DEVD', 'Gly1-Val2-Thr3-Ser4' to 'GVTS', '(Arg-Glu(EDANS)-Ser-Gln)' to 'RESQ', 'TRDI-pY-ETD-pY-pY-RK' to 'TRDIYETDYYRK', and others.

To estimate precision of text mining, 50 sequences with the combined score above the threshold for inclusion in PepBank were selected at random from the text mining output. Each of these positive predictions was manually verified, whether or not the word contained a peptide sequence (40 out of 50 were found correctly, precision = 0.8), and whether or not the word contained a peptide sequence AND the sequence was parsed 100% correctly (35 out of 50 correct, precision = 0.7). If the identified sequence was a partial protein sequence, rather than a peptide or a phage display sequence, it was considered an error: such sequences are typically entered in protein databases and do not need to be mined from text (most of the errors in precision were of this type). One or more incorrect amino acid was also considered an error.

For estimating recall, we created a separate test set of 50 sequences by searching in PubMed for recent review articles using as a query "peptide OR peptides" alone or in combination with "sequence OR sequences", and followed the PubMed abstract links for the references cited in the reviews. Peptide sequences were manually extracted from the abstracts without any automated pattern matching. The text mining output with the combined score above the threshold for inclusion in PepBank was matched against these positive real cases. Again, for each case we manually verified whether or not the algorithm found the word, which contained this peptide sequence (12 out of 50 correct, recall = 0.24), and whether or not the algorithm found the word AND the sequence was parsed 100% correctly (10 out of 50 correct, recall = 0.2). Most of the errors in recall were due to blanks (often typos) inside peptide sequences or due to unrecognized amino acid modifications.

The pioneering method to identify DNA and protein sequences in text, based on Markov models was described by Wren and co-workers [57]. Our text mining method, while similar in spirit, has different goals and thus uses a different sequence identification strategy. One of our main goals was to rapidly identify peptides with potential therapeutic and diagnostic utility (including those derived from phage display peptides), rather than identifying peptide epitopes and providing an aid to their manual curation. We also use extensive context information from the abstract, and collect peptide motifs in addition to sequences. We clean the sequences and provide access to the data for biologists through a simple web-based interface for text and sequence similarity searches. We do not place a minimum length restriction on sequences, such as 6 amino acids, because many therapeutic peptides are relatively short, for example the well-known RGD motif and many others found in phage display. Due to the substantial differences in goals and methods between our approach and that of others, it may be interesting to develop in the future a hybrid method combining the strengths of both approaches.

### Other sources

All peptide sequences with length 20 or below were extracted from ASPD [11] and UniProt [1], and fields that mapped to PepBank were parsed and stored (for example, interactor fields from ASPD, peptide fields from UniProt). The links from PepBank to the source databases were provided for all entries. Many of the peptides were stored in UniProt as part of the longer precursor proteins, producing peptides on cleavage. These peptide sequences were extracted using the UniProt feature table by selecting those with feature key "peptide" or "chain" and feature length under 20. Additional entries were manually curated, capturing the available interaction data, from the full text articles on phage display in PDF format. The articles were chosen to represent a small but diverse selection of reports within this field.

## Utility and discussion

### User interface

The web-based user interface to PepBank offers text search (both Quick and Advanced), as well as sequence similarity search (BLAST and Smith-Waterman algorithms). The Quick Search function offers a simple, Google-like search for biologists looking for peptide data in all fields. Advanced Search options include querying data by individual fields. Exact search, wildcard (*) and any single character (_) are supported in text search, which enables, for example, searching for a sequence pattern as a query. The results of the text search are displayed as a table sortable in the browser, with hyperlinks to the original sources (MEDLINE/PubMed, ASPD, UniProt) and to more detailed information.

### Text search example: VEGFR related peptides

**Figure 2:**
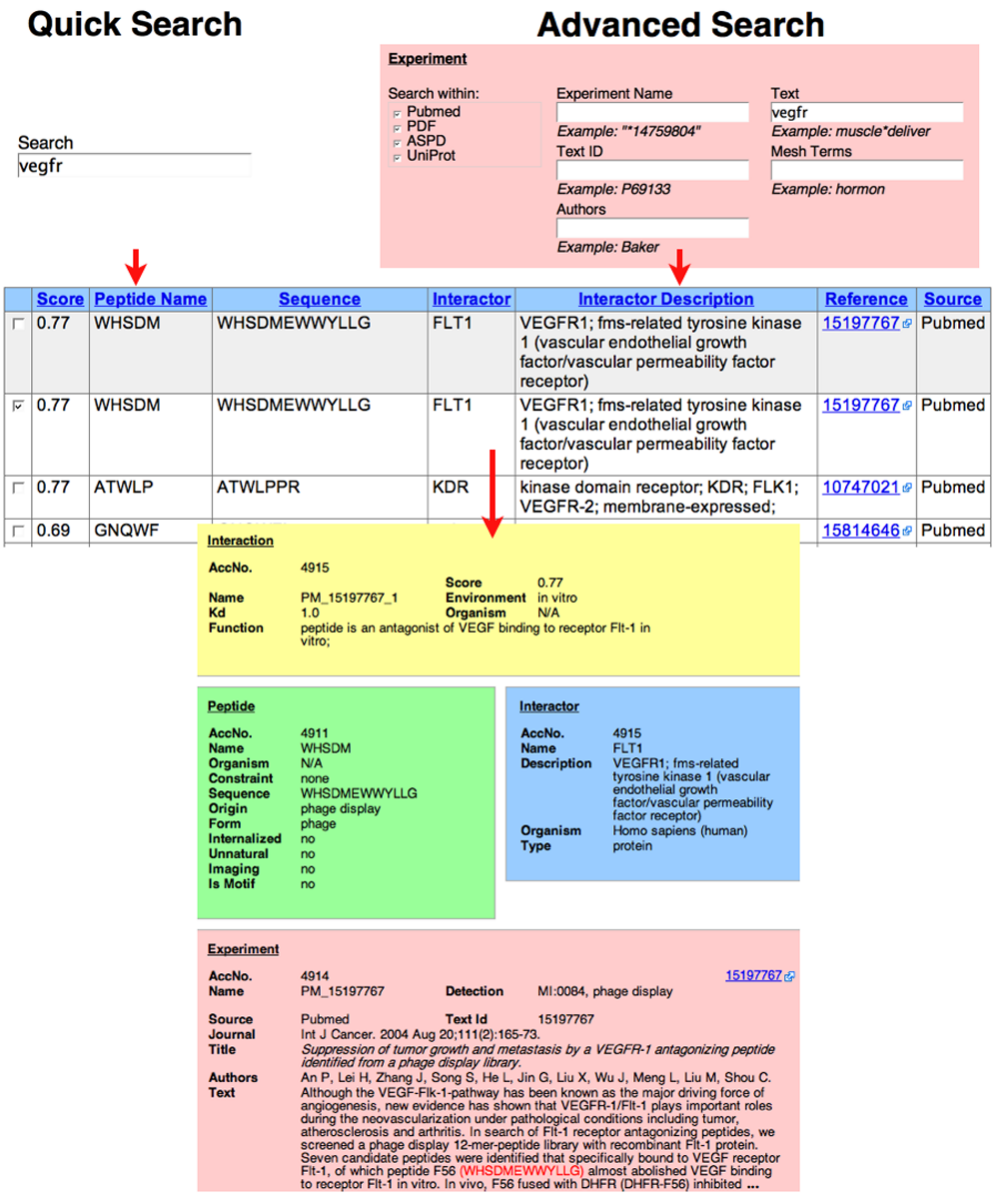
**Web-based user interface of PepBank**. Illustration of a typical user workflow. The user enters the query with Quick or Advanced Search. The results are returned in a table sortable in the browser. The user selects the entry or entries of interest. The sequence in the example shown was obtained by text mining and was then manually curated. The score, between 0 and 1, reflects the degree of confidence in the interaction (higher score for more confidence). Manually curated entries receive higher score than entries from automated text mining.

To illustrate the utility of PepBank, we use the example of identifying peptides with affinity to VEGFR1, an important therapeutic target [58]. The user can search for VEGFR using either Quick or Advanced Search, obtain a set of peptide sequences related to this target, and view details for the selected sequences. In the example shown in Figure 2, sequence 'WHSDMEWWYLLG' is identified [59]. Prompted by these results, the user of PepBank may be interested in testing this peptide sequence in novel forms (for example, dendrimers, or conjugated to nanoparticles), or for novel biomedical applications (imaging different tumor types, atherosclerosis, or arthritis). There is currently no database where the user can easily obtain such information as it relates to molecular targets and peptide sequences. One can also query directly for a biological process (such as apoptosis or angiogenesis) or for the target cell line or tissue (such as BICR-H1 or U937).

To determine whether the database would yield target leads against known drug targets, we randomly chose a set of 20 defined drug targets from the 547 approved drug target data set in DrugBank [60]. The randomly chosen drug targets were not skewed towards peptide receptors and included: squalene epoxidase, RAF proto-oncogene serine/threonine-protein kinase, muscarinic acetylcholine receptor M4, opioid mu receptor (OP3), adenosine A1 receptor, GABA transaminase, amidophosphoribosyltransferase precursor, tryptophan 5-hydroxylase 1, apoptosis regulator Bcl-2, matrix protein M2, vascular endothelial growth factor receptor 2 precursor, amiloride-sensitive sodium channel gamma-subunit, ribonucleotide reductase, cAMP phosphodiesterase, coagulation factor VIII, high affinity immunoglobulin epsilon receptor alpha-subunit precursor, retinol-binding protein I, glycine alpha 2 receptor, cytochrome P450 51, GABA-A receptor subunit *(C. elegans)*. Relevant peptides were defined as those interacting with the target or its ortholog, or modulating the function of the target, for example by acting as a competitor. Relevant peptides in our database were identified in approximately 25% of the above drug targets.

### Sequence similarity search examples

As an illustrative example, we performed an all-against-all BLAST search of PepBank sequences. One of the surprises was the discovery of an exact match to sequence 'GETRAPL' from phage display selection for peptides that bind to secreted protein acidic and rich in cysteine (SPARC) [61]. The sequence had a BLAST hit with an E-value of 0.06 to an isolate from phage display selection of peptides that bind human saphenous vein smooth muscle cells [62]. Following the BLAST results, we then found that in addition to these 2 selections, the exact same sequence was isolated independently multiple times by different groups in selections with unrelated targets. GETRAPL was found in phage display selections of peptides that bind human immunodeficiency virus type 1 (HIV-1) accessory viral protein (Vpr) [63], chromatin high mobility group protein 1, box A (HMGB1) from rat [64], mouse skeletal muscle tissue *in vivo *[65], and mouse brain cells *in vivo *[66].

We suggest that one of the utilities for PepBank is to search the peptide sequences of interest to the user with BLAST or Smith-Waterman algorithms to find any important similarities to the known peptides collected in our database. In this example, the search can be used to remove a relatively nonspecific binder GETRAPL. Note that searching PepBank with these tools is a unique resource: an exact match may be easy to find, but using a partial match such as GETRA as a query finds GETRAPL only in PepBank, but not in PubMed [2] or on Google. Searching with BLAST [67] or with Smith-Waterman/SSEARCH methods [47] using GETRAPL as a query against nr database [2] gives no peptide hits cited above. A large interactions database IntAct [6] gives no hits for GETRAPL query at all.

Another surprise discovery in the all-against-all BLAST search of PepBank sequences was the multiple occurrence of the sequence SVSVGMKPSPRP. The sequence had several exact matches over its entire length of 12 amino acids, with an E-value of 1 × 10^-6^. It was isolated in phage display selection for peptides that bind to DNA [68]. In this selection SVSVGMKPSPRP was the only sequence studied due to its dominance (9 out of 10) in the selected pool. The exact same sequence was isolated in phage display selection for peptides binding to human monoclonal IgM [69], and to the mirror image of Alzheimer's disease amyloid peptide Abeta(1–42) [70]. The sources for these sequences were MEDLINE abstract text mining, ASPD database, and manually curated full text articles, respectively. In addition, SVSVGMKPSPRP occurs in several patents [71,72]. Several groups note multiple isolation of this remarkable sequence in their own and other, unrelated, experiments [73,74]. The sequence has also been identified in a recent excellent review [24] which covers the important topic of target-unrelated sequences in phage display. Interestingly, all of the studies with both GETRAPL and SVSVGMKPSPRP were done with the phage display libraries from the same manufacturer, thus suggesting a library- or methodology-specific phenomenon. Both sequences illustrate one of the suggested utilities for PepBank, namely that one can search it with a sequence query using BLAST or Smith-Waterman algorithms to find any important similarities to the known peptides.

## Conclusion

A new text mining tool was developed and used to identify peptide sequences in MEDLINE abstracts. These data were combined with two of the public sources of peptide sequence data, ASPD and UniProt, as well as with manually curated peptide data. The database application was developed to query the data using text and sequence similarity search through a web-based user interface. The utility of PepBank was demonstrated using different examples of peptide sequences. The results show that the database has valuable biological and medical applications. In the future, we plan to add other public sources of peptide data, such as the peptide subset of the Molecular Interaction database (MINT) [5], and other sources for text mining, such as full-text journal articles. Also, in the future we will apply machine learning techniques to improve the accuracy of text mining to extract sequences. In the next release, we plan to add the functionalities to download the data in a standard format, such as PSI MI, and to search the database for peptide motifs.

## Availability and requirements

The database is freely available on , and the text mining source code (Peptide::Pubmed) is freely available above as well as on CPAN .

## Authors' contributions

TS designed and developed the text mining algorithm, curated the database contents, co-designed the database and the interface and wrote the manuscript, DG designed and developed the database, the web application and the interface, MK co-curated the database contents, MP designed the architecture of the entire web site and designed the database and the interface, RW provided the conceptual design and the overall guidance of the entire project and co-wrote the manuscript. All authors read and approved the final manuscript.
